# The Selective Myosin II Inhibitor Blebbistatin Reversibly Eliminates Gastrovascular Flow and Stolon Tip Pulsations in the Colonial Hydroid *Podocoryna carnea*

**DOI:** 10.1371/journal.pone.0143564

**Published:** 2015-11-25

**Authors:** Noah Connally, Christopher P. Anderson, Jules E. Bolton, Edward W. Bolton, Leo W. Buss

**Affiliations:** 1 Department of Ecology and Evolutionary Biology, Yale University, New Haven, Connecticut, United States of America; 2 20 Colony Road, New Haven, Connecticut, United States of America; 3 Department of Geology and Geophysics, Yale University, New Haven, Connecticut, United States of America; 4 Smithsonian Marine Station, Fort Pierce, Florida, United States of America; UC Irvine, UNITED STATES

## Abstract

Blebbistatin reversibly disrupted both stolon tip pulsations and gastrovascular flow in the colonial hydroid *Podocoryna carnea*. Epithelial longitudinal muscles of polyps were unaffected by blebbistatin, as polyps contracted when challenged with a pulse of KCl. Latrunculin B, which sequesters G actin preventing F actin assembly, caused stolons to retract, exposing focal adhesions where the tip epithelial cells adhere to the substratum. These results are consistent with earlier suggestions that non-muscle myosin II provides the motive force for stolon tip pulsations and further suggest that tip oscillations are functionally coupled to hydrorhizal axial muscle contraction.

## Introduction

*Podocoryna carnea*, on which this study is based, resembles the vast majority of encrusting colonial hydrozoans in adhering to and advancing over the substratum by stolons [1]. The stolonal network, known as the hydrorhiza, extends by elongation of stolon tips, establishment of new tips by lateral branching and is supported by feeding polyps that bud atop stolons [2–4]. All tissues are diploblastic and the gastric cavity of polyps is coextensive with the lumen of stolons [4]. This internal fluid conducting system is known as the gastrovascular system [5,6]. While the entire colony is sessile, tissues do move.

Two movements with different functions are known. When fed, a polyp begins a series of contractions that act to drive fluids into the gastrovascular system where nutrients are subsequently absorbed [7–11]. Another set of movements characterize stolon tips and polyp buds. These tissues are histologically identical at early stages and both elongate by a series of pulsations, where the rudiment extends and retracts in an oscillatory fashion, with the net movement being positive in growing colonies [12–22].

The cell biology of these movements is little studied. However, two recent investigations suggest a prominent role may be played by non-muscle myosin II (NMII). Steinmetz, et al [23] documented the expression of muscle and non-muscle myosin in the polyp of the anthozoan *Nematostella vectensis* and in the medusa of the hydrozoan *Clytia hemasphaerica* and found that NMII was expressed in all gastrodermal epitheliomuscular cells, where circular smooth muscle fibers are located. Buss et al [24] showed that the gastrodermal cells of *P*. *carnea* hydrorhiza possess smooth muscle fibers that serve to expand and contract the stolonal lumen and that this axial musculature is continuous with the circular muscles of the polyp. Concatenating the findings of these two studies led us to predict that inhibition of NMII will disrupt gastrovascular flow.

Non-muscle myosin II has also been predicted to play role in stolon tip pulsations [24]. Stolon tips are capped by adherens junctions [24], which serve a contractile function motored by NMII in a variety of systems [25]. In addition to adherens junctions, elongation of tips requires movement of cells along the mesoglea. Tips, moreover, must adhere to the surface and extend along it. Cell movements along a basement membrane and extension of cells by focal adhesions are likewise dependent on NMII in bilateral model organisms [26]. One might reasonably predict that tip pulsation would be silenced by inhibition of NMII.

We here put these two predictions to an experimental test and find that blebbistatin reversibly eliminates both tip pulsations and gastrovascular flow.

## Methods

### Animal Care

**Fig 1:**
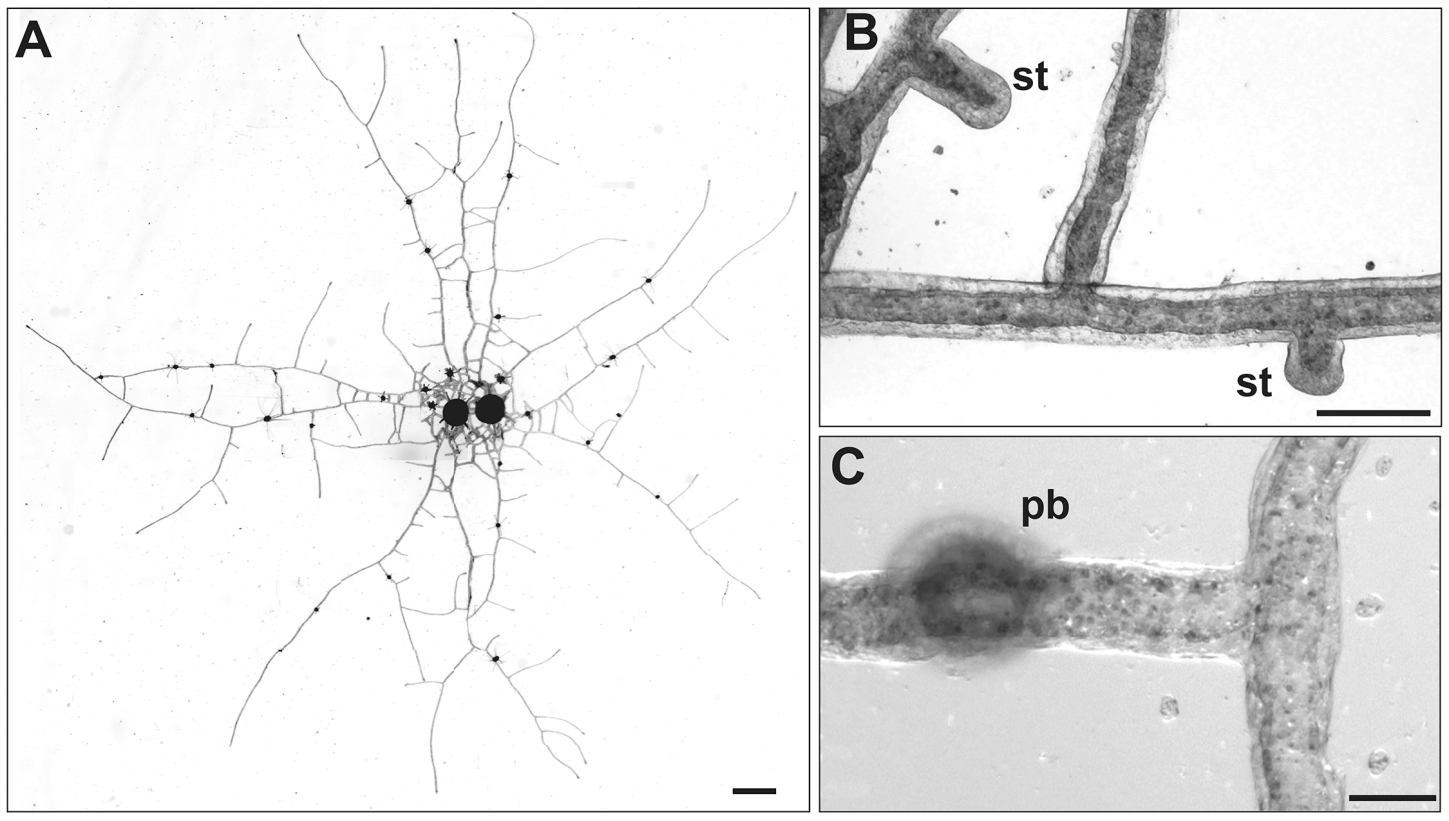
*Podocoryna carnea* (A) Top view of a young colony. Scale bar: 1 mm. (B) Small region of hydrorhiza showing elongating stolon tips (st). Scale bar: 100 μm. (C) Stolon bearing a polyp bud (pb). Scale bar = 50 μm.

*Podocoryna carnea* is an athecate hydroid that forms an extensive, essentially two-dimensional hydrorhizal network (Fig 1A). The species is typically found as an epibiont on hermit crab shells in nature and is readily propagated in the laboratory. Colonies grow by elongation and lateral branching of stolons and the formation of polyp buds atop existing stolons (Fig 1B and 1C).

Our studies employ colonies of a single strain (P3) of *P*. *carnea*, collected from the intertidal of Lighthouse Point, New Haven CT in 1989 and propagated asexually in the laboratory ever since. Collections were made under a permit issued by the Connecticut Department of Environmental Protection. *Podocoryna carnea* is neither a protected nor an endangered species.

Colonies were maintained under standard conditions [27]. Briefly, colonies are grown on glass microscope slides or glass cover slips. Clonal replicates are generated by explanting a small region of the hydrorhiza bearing 1–3 polyps and affixing them to the glass surface with a loop of quilting thread. After 2 days the colonies have attached and the thread is removed. Colonies are held in recirculating aquaria with daily exchanges of 25% of the seawater (31 ppt). Colonies are fed to repletion every other day with 3–4 day old *Artemia salina* nauplii. All experiments described here were performed on animals that had been fed one day earlier.

### Imaging

Digital images of stolon tips were obtained using a Zeiss Axiovert 135 compound microscope and Zeiss Axiovision image acquisition software. Colonies growing on cover slips were observed in a stage-mounted, flow-through culture system described earlier [28]. Briefly, the flow-through system utilizes a Warner RC-50 Ussing chamber modified to accommodate 22 x 40 mm cover slips as top and bottom to create a closed bath with an internal volume of 4 ml. Water is continuously circulated through the chamber using a Harvard Apparatus PHD 2000 push/pull infusion pump fitted with 4 x 50 ml syringes at a rate of 5 ml/minute. Stolon tips were imaged at 400X using differential interference contrast microscopy. Images were acquired at 4 or 8 second intervals.

### Reagents

Blebbistatin was identified in a small molecule screen designed to identify reagents that block NMII [29]. In vertebrates, three isoforms of NMII are known and all are inhibited by blebbistatin. Muscle myosin II has also diversified into a number of isoforms in vertebrates and some of these are can be inhibited by blebbistatin [29–31]. The effect of blebbistatin on cnidarian muscle has not been previously explored. We also tested the response of stolon tips to latrunculin B, which sequesters G actin preventing F actin assembly [32].

Two carrier solutions were prepared: one with 0.15 μl/ml DMSO in filtered seawater and the other with 0.09 μl/ml DMSO. These carrier solutions served as the DMSO control. Blebbistatin (0.255 mM) was prepared in the carrier solution of 0.15 μl/ml dimethyl sulfoxide (DMSO) and latrunculin B (5μM) in carrier solution of 0.09 μl/ml DMSO. All experiments began with imaging of the stolon tip in the DMSO control solution for 60–90 minutes. Following this period the water supply was switched to either blebbistatin for 90 minutes or latrunculin for 9 minutes. Following the incubation period, the water supply was returned to the DMSO control solution and the colony continuously imaged for up to 6 hours thereafter. Photoinactivation was prevented in the blebbistatin experiments by use of a red filter (Lee #026, >580 nm) [33,34]. One replicate of the stolon tip experiment was inadvertently exposed to blebbistatin for 81 rather than 90 minutes.

We tested whether contraction of epithelial longitudinal muscles were inhibited by blebbistatin by treating colonies with blebbistatin as described above and thereafter exposing polyps to a brief pulse of 0.1M KCl.

### Analysis

**Fig 2:**
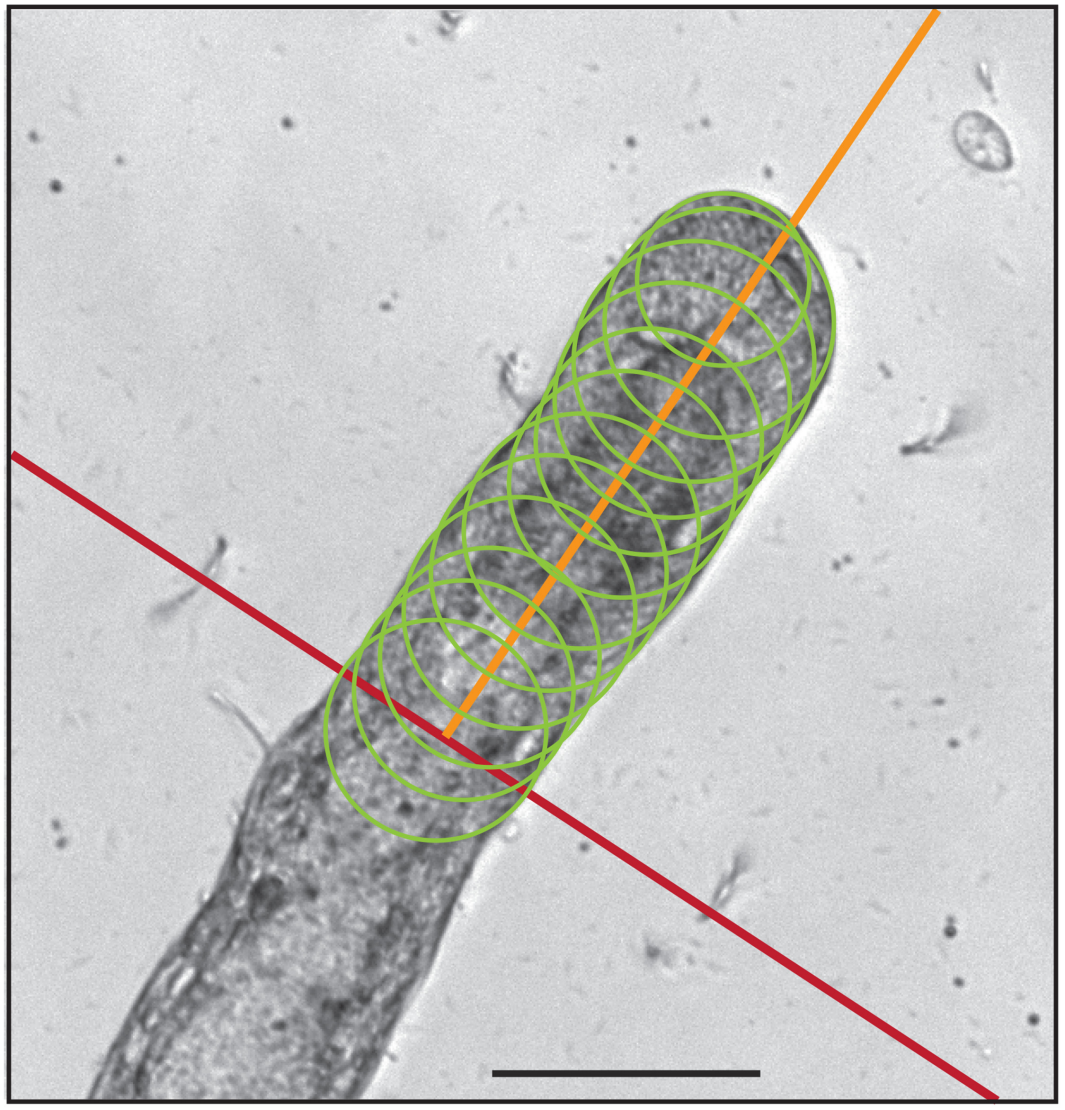
Procedure for automated measurement of stolon length. Algorithm described in text and R code provided in S1 Software. Scale bar: 50 μm.

Measurement of stolon tip pulsations from images was automated by use of a custom R code (S1 Software). On the initial image of the time-series, the user sets a reference line (red line, Fig 2) from which to measure the growth and an axis (orange line, Fig 2) along which the growth will be measured. The program draws the largest circle on the red line for which 99.85% of the pixels are within the stolon's area (green, Fig 2). Similar circles are generated for cross sections parallel to the red line, going down the axis of growth. These areas are combined to estimate the location and area of the stolon. From this bounded area, length along the axis of growth is obtained.

**Fig 3:**
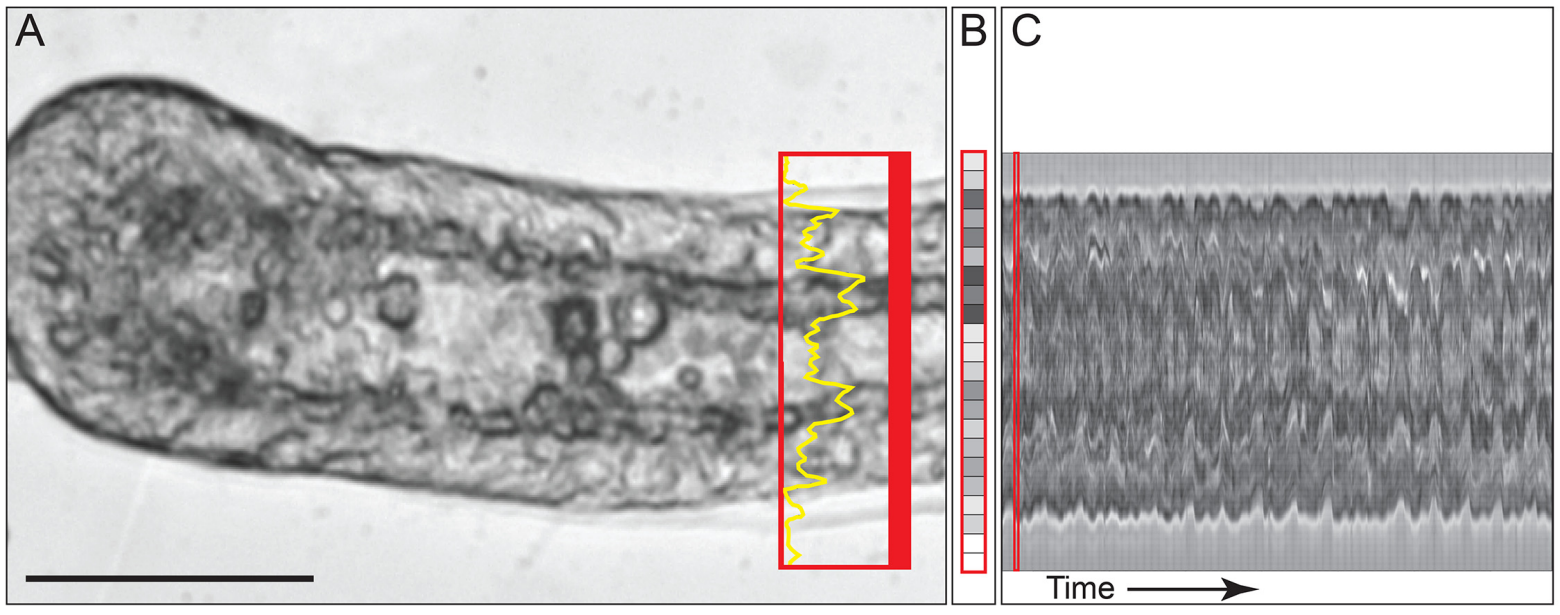
Procedure for visualizing stolon lumen oscillations. Algorithm described in text and R code provided in S2 Software. Scale bar: 50 μm.

Visualization of the lumen oscillations was facilitated by another custom R code (S2 Software). The user selects a 30-pixel region along the proximal-distal axis of the stolon in which to measure the cross-lumen luminosity time series (broad red bar, Fig 3A). The average grey-value is acquired using tools in Zeiss Axiovision software (yellow line, Fig 3A), which allows the output of such profile time series as 2^k^ binned grey values with rows indicating x-y locations (where k = 1,2, …8), and columns representing time. The R code then reads this data, takes the transpose of the array of grey values, and then images the profiles in time and cross section location, creating a kymograph of the movement of the lumen over time (Fig 3C).

We quantified epidermal cell movements at the stolon tip from films in which the lateral margins of tip epidermal cells were clearly identifiable. To analyze these images, we located the distal-most point on the stolon tip and selected two additional locations, one basal and the other apical, on the lateral margin of a single epidermal tip cell. For 16 minutes, we recorded every 8 seconds whether each of these points moved in the distal or proximal direction, or whether the point remained stationary.

## Results

**Fig 4:**
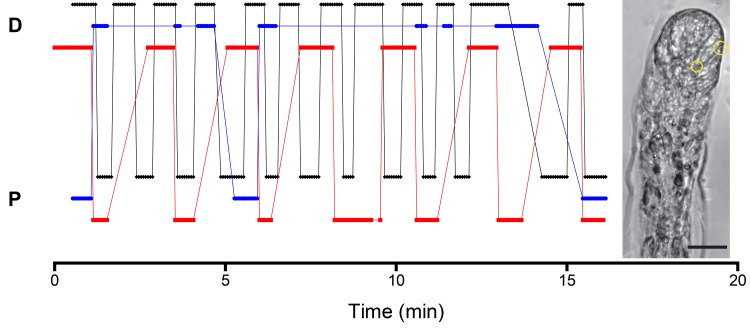
Distal (D) and proximal (P) movement of the stolon tip (black) and of apical (blue) and basal (red) epidermal cell surfaces. Vertical offsets introduced to allow visualization of overlapping data points and do not signify differing extent or rate of movement. Absence of a data point at any time interval does not signify missing data. Rather, at these time points no motion was detected between that time point and the time point preceding it. Inset shows location of apical and basal measurements. Scale bar: 20 μm. The images from which the data was acquired are displayed in S1 Movie.

Tip epidermal cell movements are shown in S1 Movie and quantified for a selected epidermal cell in Fig 4. The tip and the basal region of an epidermal cell undergo regular dorsal extensions and proximal retractions. These oscillations are not in synchrony. The tip oscillates at the higher frequency. In contrast, apical region of the epidermal cells rarely moved in the proximal direction, but most frequently remained stationary, punctuated by distal-ward movements that varied in frequency and duration. Apical and basal regions of same lateral cell margin are typically, but not always out-of-phase.

**Fig 5:**
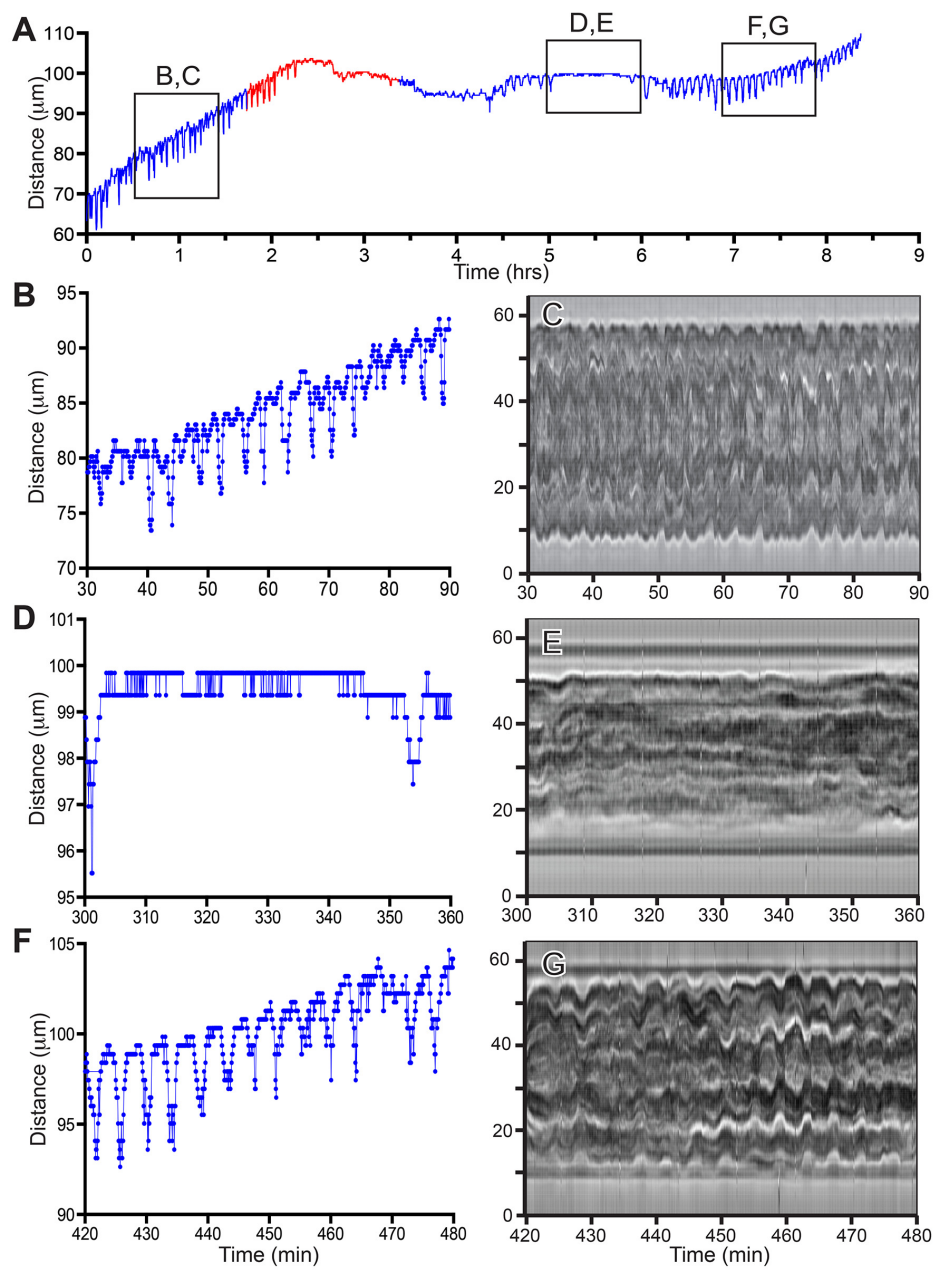
Intact stolon. (A) Tip elongation before and after (blue) blebbistatin treatment (red). Frame rate: 4 seconds, n = 7536. (B, D, F) Tip elongation in selected time intervals (boxes in A). (C, E, G) Behavior of gastrovascular lumen in the same time intervals as (B, D, F).

The effect of blebbistatin on one stolonal tip is shown in Fig 5 and S2 Movie. An interval was identified during which blebbistatin eliminated all tip pulsations (Fig 5A and 5D). The effect is reversible (Fig 5F), but not immediate. The application of blebbistatin also had the effect of reversibly eliminating all gastrovascular lumen oscillations (Fig 5C, 5E and 5G).

This experiment was repeated six times (Table 1). Blebbistatin disrupted tip pulsations and lumen oscillations in all cases (Table 2). Tip pulsations were silenced in 5 of 6 replicates. In the case where elongation continued, blebbistatin depressed the growth rate of the tip by factor of 2.4. Regular lumen oscillations ceased in all replicates, although in four of the six replicates occasional lumen contractions of variable amplitude were observed at irregular intervals. Resumption of tip pulsations preceded return of regular lumen oscillations. The effect of blebbistatin on growth rate and period were highly significant (Table 2).

**Table 1:** Summary Statistics for Replicate Colonies.

	Blebbistatin									Latrunculin		
	Intact Colonies						Isolated Stolons						
Replicate #	1	2 ^a^	3^b^	4	5	6	1 ^c^	2	3	1 ^d^	2 ^e^	3
Time (min) at which tip pulsations stop after reagent addition	42	34	104		74	129	69	57	58	6	7	6
Time (min) at which lumen oscillations stop after reagent addition	30	32	18	32	56	60	64	55	48	1	2	3
Time (min) at which tip pulsations resume after reagent removal	53	154	42		78	77	43	51	84	55	14	113
Time (min) at which lumen oscillations resume after reagent removal	148	207	71	35	231	88	19	49	190	39	32	55
Growth rate (μm/hr) prior to reagent addition	11.6	30.8	16.2	47.3	24.0	44.3	16.6	18.7	6.8	14.5	27.2	51.1
Growth rate (μm/hr) after reagent addition	0	-3.4	0	19.9	0	0	2.9	1.4	0	-136.1	-31.7	-86.2
Growth rate (μm/hr) after pulsations resume	8.6	17.4	10.4	37.3	18.5	8.8	13.8	22.8	22.8	9.3	24.5	36.4
Lumen period (sec) prior to reagent addition^f^	225	225	415	245	270	284	235	360	450	225	225	225
# Contractions/min after reagent addition^f^	0.07	0.01	0	0.09	0	0.02	0.09	0.02	0.02	0	0	0
Lumen period (sec) after flow resume^f^	257	284	415	338	257	338	270	491	270	180	450	225

^a^ Time-series presented in Fig 5, S2 Movie

^b^ Colony exposed to reagent for 81 rather than 90 minutes

^c^ Time-series presented in Fig 6, S3 Movie

^d^ S4 Movie

^e^ Time-series presented in Fig 7

^f^ Measured over 90 minute interval in blebbistatin treatments and 15 minute intervals in latrunculin treatments

**Table 2:** Student’s t-test, p values^a^.

	Growth Rate		Period	
	Pre-treatment versus Treatment	Treatment versus Recovery	Pre-treatment versus Treatment	Treatment versus Recovery
Intact Colony	0.0034	0.0319	<0.0001	<0.0001
Isolated Stolon	0.0286	0.0041	0.0052	0.0098
Latrunculin	0.0225	0.0256	N/A^b^	N/A

^a^ Computed from Table 1

^b^ Mean = standard deviation = 0 for the treatment, which precludes the use of the t-test

Gastrovascular flow is driven by polyps. We were curious what might arise in an isolated stolon and so severed stolons from colonies. The cut margin sealed immediately and both tip and lumen oscillations ceased. Fifteen isolated stolons were produced (length: 333 +/- 69 μm), each with one intact tip and one severed tip. After a period of hours, a new tip forms at the cut end and begins to elongate. In 12 of 15 trials, only the severed end elongated. In the remaining 3 trails, both tips pulsated and neither grew appreciably as might be expected by the fact that the number of cells was neither increasing nor decreasing.

**Fig 6:**
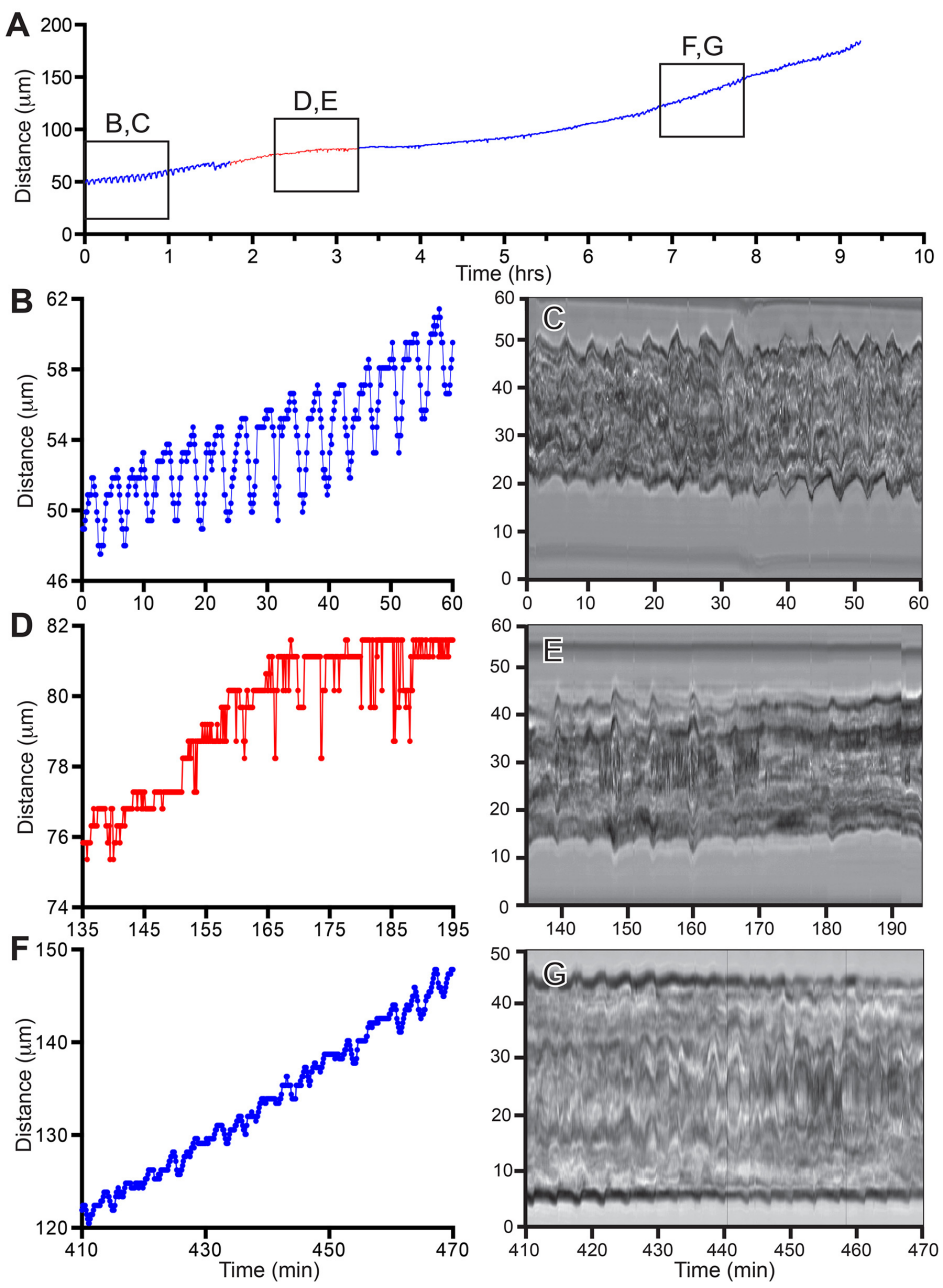
Isolated Stolon. (A) Tip elongation before and after (blue) blebbistatin treatment (red). Frame rate: 8 seconds, n = 4161. (B, D, F) Tip elongation in selected time intervals (boxes in A). (C, E, G) Behavior of gastrovascular lumen in the same time intervals as (B, D, F).

The effect of blebbistatin on a severed tip is shown in Fig 6 and S3 Movie. Blebbistatin reversibly eliminated tip elongations and disrupted gastrovascular lumen oscillations. This experiment was repeated three times (Table 1). In all cases, both tip pulsations and gastrovascular flow were reversibly eliminated. As in the case of intact colonies, the effects of blebbistatin on growth rate and period were highly significant (Table 2).

**Fig 7:**
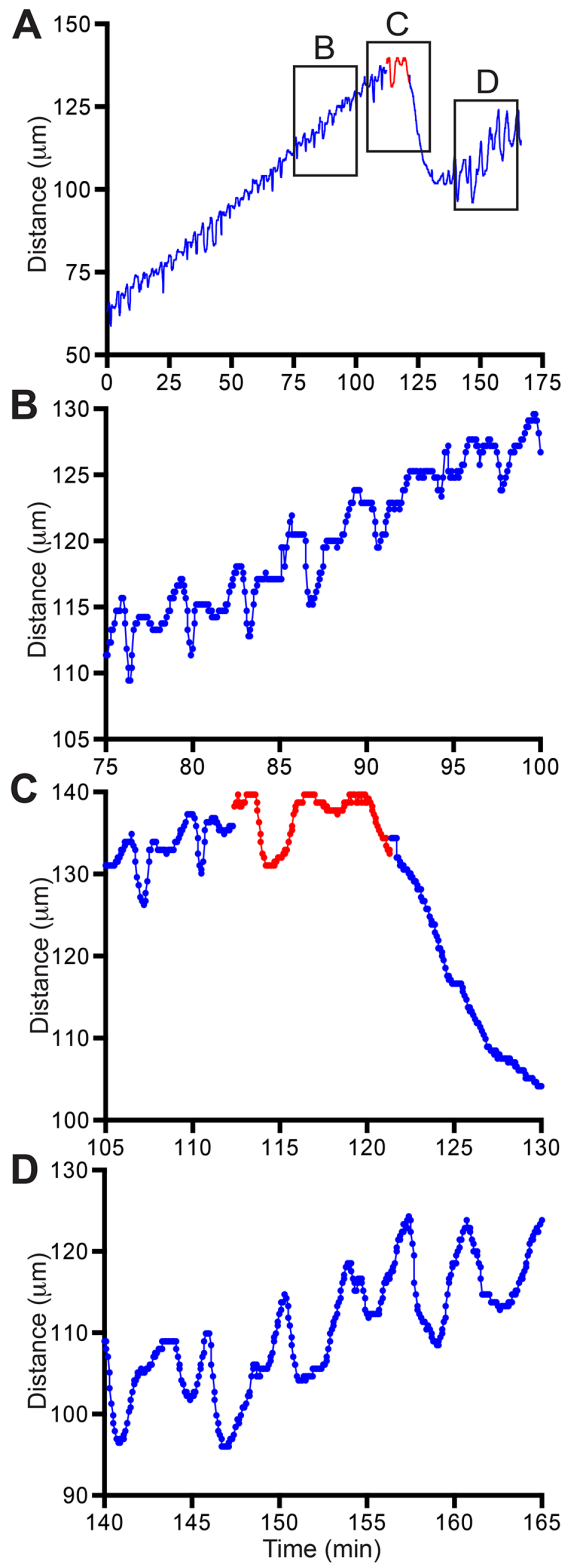
Response of intact stolon to latrunculin. (A) Tip elongation before and after (blue) blebbistatin treatment (red). Frame rate: 8 seconds, n = 2496. (B-D) Tip elongation in selected time intervals (boxes in A).

**Fig 8:**
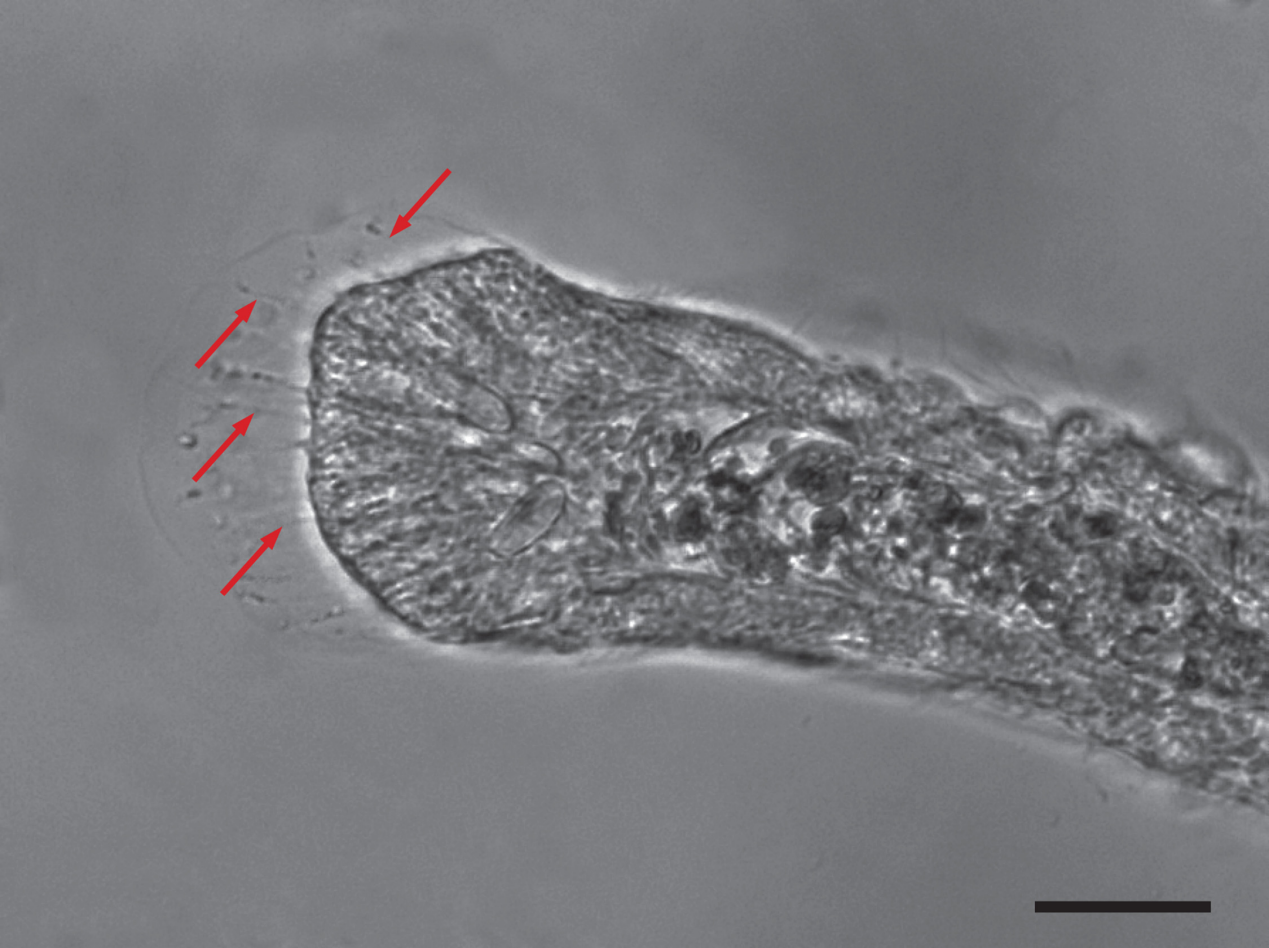
Focal Adhesions. Latrunculin induces retraction revealing actin filaments (arrows). Time: 7:52 minutes:seconds after latrunculin addition. Scale bar: 20 μm. Image from S4 Movie.

Application of latrunculin to elongating stolon tips results in an immediate and pronounced retraction of the stolon tip and cessation of all tip elongation (Fig 7). The effect was reversible. The retraction revealed extensions of tip epidermal cells resembling focal adhesions, within which actin filaments can be visualized (Fig 8, S4 Movie). This experiment was repeated three times (Table 1). The effects of latrunculin on growth rates were significant (Table 2).

Epithelial longitudinal muscles of polyps were not inhibited by blebbistatin. All polyps exposed to a pulse of KCl exhibited contraction along the oral-aboral axis (n = 13).

The time-series data and raw images for the experiments shown in Figs 5–7 and for analyzed intervals of all replicates (Table 1) are available at the Dryad Data Repository (DOI:10.5061/dryad.40494).

## Discussion

### Tip Pulsations

Extensive descriptions of the cell movements associated with stolon tips and polyp buds are available for a number of thecate hydroids [16,20–22]. Our observations of the epidermal cell movement in the stolon tip of the athecate *Podocoryna carnea* are in broad accord with their findings. Specifically, our results concur with earlier findings that the stolon tip periodically extends and retracts and that these movements are associated with periodic shifts in the relative positions of apical and basal regions of tip epidermal cells. We likewise find that distal extensions of the apical regions of a tip epidermal cell are typically associated with proximal movements of the basal regions of that cell. Our results differ from earlier findings in that tip pulsations in *Podocoryna carnea* are more variable than that described for thecate hydroids. In particular, we find that the periods of tip oscillations exceeds that of oscillations in the position of basal regions of epidermal cells and that retractions of the apical regions of epidermal cells occur infrequently.

Blebbistatin reversibly eliminated or suppressed tip pulsations in both the epidermis and gastrodermis of stolon tips. The effect of blebbistatin was reversible, but recovery was not immediate. The pace of recovery was surprising, since recovery in studies of bilaterian systems is rapid [26]. We suspect this finding reflects the fact that stolon tips in athecate hydroids are not continuously active [11,35], which implies the existence of a mechanism governing whether a tip is active or inactive. We hypothesize that a tip once silenced may require some latency period before resuming activity.

Our experiments do not identify the sites at which the blebbistatin-induced inhibition is acting. However, several hypotheses are easily constructed. First, the distal end of stolon tips are bound in adherens junctions [24], which have a NMII mediated contractile function in a diversity of systems [26]. Such contractions may be associated with the rapid distal extension of apical region of tip epidermal cells noted in our work (Fig 4) and in prior study of tip cell movements [16,20–22]. NMII has also been implicated in the migration of cells along the basement membrane, typically by integrin linkages to the actin cytoskeleton [36]. Actin filaments have been localized in basal regions of both gastrodermal and epidermal tip cells [24] and inhibition of NMII may act at these sites as well. Finally the tip epidermis must extend across and adhere to the substratum. Application of latrunculin revealed focal adhesions where epidermal cells protrude along the substratum. These protrusions are motored by NMII in axons and in fibroblasts migrating on culture dishes [37]. Apical and basal movements are necessarily mechanically coupled both within and between cells, as are movements of the epidermis and the gastroderm. Tip oscillations are, thus, a complex mechanical system for which the underlying cell biology remains largely unexplored. The ease of *in vivo* experimentation using colonial hydroids recommends them as a useful model for the study of collective cell movements.

### Gastrovascular flow

Our experiments show that blebbistatin eliminated periodic gastrovascular flow in all replicates. The observation that gastrovascular flow was entirely eliminated in isolated stolons is particularly germane. The only muscles in stolons are the gastrodermal smooth axial muscles [24], which are continuous with the circular gastrodermal smooth muscles of the polyps. Thus, this result provides an experimental demonstration that blebbistatin inhibits these muscles. What is less clear, however, is whether this inhibition is attributable to NMII or to inhibition of muscle myosin II. A circumstantial case can be made for NMII. NMII is expressed and muscle myosin II not expressed in the gastrodermal cells of the *Nematostella vectensis* polyp and the *Clytia hemasphaerica* medusa [23], cells which are characterized by smooth muscle fibers. Moreover, studies of mice in which smooth muscle myosin II has been knocked out have established that smooth muscle contraction can occur using NMII [38,39]. We cannot, however, conclude that *Podocoryna* axial muscles are motored by NMII on the current evidence because contraction of vertebrate arterial smooth muscle myosin II have also been shown to be inhibited by blebbistatin [31]. This question would be clarified by studies of the expression of the NMII and muscle myosin II in the *Podocoryna* hydrorhiza.

The observation that gastrovascular flow was observed in the isolated stolon experiments is of importance for a second reason. In intact colonies gastrovascular flow is driven by the contractions of polyps [6,8,11]. The fact that severed stolons, which lack polyps, can also drive gastrovascular flow and that the onset of this flow follows the onset of tip pulsations is consistent with prior suggestions that tip pulsations trigger stolonal axial muscles [11,24]. Communication between tips and axial muscles is presumably neuronal, as nerves have recently been localized to proximal regions of the stolonal tip gastroderm [24]. While it has long been known that, in intact colonies, polyp-driven gastrovascular oscillations are not in synchrony with tip pulsations [16,19,22,40], our observations suggest a functional coupling of the two movement systems via the neuromuscular system. This possibility is ripe for further exploration.

## Supporting Information

S1 Movie Movement of epithelial cells at stolon tip. Time in hours:minutes:seconds. Arrows indicate selected cell margins. Frame interval: 8 seconds. Scale bar: 20 μm.
(MOV)

S2 Movie Intact stolon tip during the three time intervals shown in Fig 5. Time in hours:minutes. Frame interval: 4 seconds. Scale bar: 20 μm.
(MOV)

S3 Movie Isolated stolon tip during the three time intervals shown in Fig 6. Time in hours:minutes. Frame interval: 8 seconds. Scale bar: 20 μm.
(MOV)

S4 Movie Response of stolon tip to latrunculin. Time in hours:minutes. Frame interval: 4 second. Scale bar: 20 μm.
(MOV)

S1 Software R script used to calculate stolon length. (PDF)

S2 Software R script used to generate kymographs. (DOCX)
