# Supplementary material for: The Selective Myosin II Inhibitor Blebbistatin Reversibly Eliminates Gastrovascular Flow and Stolon Tip Pulsations in the Colonial Hydroid Podocoryna carnea
Source: PLoS One. 2015 Nov 25;10(11):e0143564. doi: 10.1371/journal.pone.0143564 (PMC4659590; doi:10.1371/journal.pone.0143564)
Supplement: S1 Software — (PDF) [file pone.0143564.s005.pdf]

This R code creates a GUI that allows the user to track the growth of
stolon tips over a series of .tiff images (frames). After copying this
entire text into the R console, the user launches the program using
the R command `begin()`. The user is then prompted to crop,
correctly orient, and clean the first image in the time series. Next,
the same steps are automatically applied to all other images of the
time series. The growth of the stolon over time is saved to `stats`.
Each image frame with points indicating the border of the stolon
and the length and direction of growth is saved as `day.jpg`.

If an instruction window appears, the user must close the box
clicking ‘OK’ before proceeding.

Set your working directory to the folder containing your .tiff images
using `setwd(‘***’)`

```
library(tcltk)
```

The following are R functions called by the program

Custom image function

```
customimage = function(mat, main, destroy, resred) {

  if(!exists('firstrun')) {

    if(destroy==F) quartz(height = 8, width = 8)

    n = ceiling(mean(nrow(mat), ncol(mat))/600)

    imagemat = matrix(0, nrow = floor(nrow(mat)/n),
 ncol = floor(ncol(mat)/n))

    for(i in 1:nrow(imagemat)) {
      for(j in 1:ncol(imagemat)) {
        imagemat[i,j] = mat[n*i,n*j]}}

    image((1:nrow(imagemat))*mpp*resred*n,
 (1:ncol(imagemat))*mpp*resred*n, imagemat,
    xlab = 'Microns', ylab = 'Microns', col =
 gray((1:256)/256), main = main)
```

```
    assign('firstplot', F, env = .GlobalEnv)}}
```

Custom locator function
This function prompts the user to click the plotting window n times.
With each click, the row and column of the imaged matrix are saved
in a new matrix called `loc`.

```
myloc = function(n, mat, style) {

  loc = matrix(nrow = n, ncol = 2)

  for(i in 1:n){

    point = locator(1)

    showpoint = T

    if(point$x > nrow(mat)*mpp*resred) {point$x =
(nrow(mat)-1)*(mpp*resred); if(style != 1) showpoint =
F}
    if(point$x < 0) {point$x = 0; if(style != 1)
showpoint = F}
    if(point$y > ncol(mat)*mpp*resred) {point$y =
(ncol(mat)-1)*(mpp*resred); if(style != 1) showpoint =
F}
    if(point$y < 0) {point$y = 0; if(style != 1)
showpoint = F}

    if(point$x==nrow(mat)*mpp*resred||point
$x==mpp*resred||point$y==ncol(mat)*mpp*resred||point
$y==mpp*resred) style = 0

    if(showpoint == T || style == 1) {

      if(style==1) points(point$x, point$y, pch = 3,
col = 'green', cex = 2)
      if(style==2) points(point$x, point$y, pch = 1,
col = 'green')
      if(style==3) points(point$x, point$y, pch = 1,
col = 'yellow', cex = 2)
```

```
      if(style==4) points(point$x, point$y, pch = 1, 
 col = 'green', cex = 2) }

    loc[i, 1] = round((point$x/mpp/resred)+1)
    loc[i, 2] = round((point$y/mpp/resred)+1)}

  assign('loc', loc, env = .GlobalEnv)}
```

Find rows and columns
This function creates two vectors `rows` and `columns` that contain
the rows and columns of all positive values in matrix `mat`.

```
mkrowcol = function(mat) {

  rows = which(mat>0)%%nrow(mat)
  cols = ceiling(which(mat>0)/nrow(mat))
  rows[rows==0] = nrow(mat)
  cols[cols==0] = ncol(mat)

  assign('rows', rows, env = .GlobalEnv)
  assign('cols', cols, env = .GlobalEnv)}
```

Find Angle
This function returns the angle (in radians) created by two points,
`p1` and `p2`.

```
findangle = function(p1, p2) {

  if((p2[1] - p1[1]) >= 0) return(atan((p2[2] - 
 p1[2]) / (p2[1] - p1[1]))%%(2*pi))

  if((p2[1] - p1[1]) < 0) return(pi + atan((p2[2] - 
 p1[2]) / (p2[1] - p1[1])))}
```

Convert to x digits
This function is used for finding the correct names of image files.
In the case where there are 1000 images, `tomaxdigs(1)`
would return "`0001`".

```
tomaxdigs = function(n) {
```

```
  digs = ceiling(log10(num2+1))

  output = ''
  d = 1

  while(d <= digs) {

    if(d == digs) return(paste(output, n%%10, sep =
''))

    if(d < digs) output = paste(output, floor(n/
(10^(digs-d))), sep = '')

    n = n - floor(n/(10^(digs-d)))*10^(digs-d)
    d = d + 1}}
```

This function opens the GUI. In the first window, the user is prompted
to enter information on the size, magnification, location, and number
# of images in the time series.

```
begin = function() {

  tt = tktoplevel()

  name = tclVar('filename base')
  ext = tclVar('extension')
  firstnum = tclVar('number of first image')
  lastnum = tclVar('number of last image')
  size = tclVar('number of bytes')
  xdim = tclVar('xdim')
  ydim = tclVar('ydim')
  path = tclVar('directory path')
  mpp = tclVar('microns per pixel')

  entryname <-tkentry(tt,width='20',textvariable=name)
  entryext <-tkentry(tt,width='20',textvariable=ext)
  entrynum1 <-
tkentry(tt,width='20',textvariable=firstnum)
  entrynum2 <-
tkentry(tt,width='20',textvariable=lastnum)
  entrysize <-tkentry(tt,width='20',textvariable=size)
```

```
  entryxdim <-tkentry(tt,width='20',textvariable=xdim)
  entryydim <-tkentry(tt,width='20',textvariable=ydim)
  dirpath <-tkentry(tt,width='20',textvariable=path)
  entrympp <-tkentry(tt,width='20',textvariable=mpp)

  tkgrid(tklabel(tt,text='   Please enter the following
image information and press OK'   ))
  tkgrid(entryname)
  tkgrid(entryext)
  tkgrid(entrynum1)
  tkgrid(entrynum2)
  tkgrid(entrysize)
  tkgrid(entryxdim)
  tkgrid(entryydim)
  tkgrid(dirpath)
  tkgrid(entrympp)

  OnOK <- function() {
    assign('namebase', tclvalue(name), env
= .GlobalEnv) #Can probably remove this stuff
    assign('nameext', tclvalue(ext), env = .GlobalEnv)
    assign('num1', as.numeric(tclvalue(firstnum)), env
= .GlobalEnv)
    assign('num2', as.numeric(tclvalue(lastnum)), env
= .GlobalEnv)
    assign('bytes', as.numeric(tclvalue(size)), env
= .GlobalEnv)
    assign('imxdim', as.numeric(tclvalue(xdim)), env
= .GlobalEnv)
    assign('imydim', as.numeric(tclvalue(ydim)), env
= .GlobalEnv)
    assign('oldpoint', c(0, 0), env = .GlobalEnv)
    assign('mpp', as.numeric(tclvalue(mpp)), env
= .GlobalEnv)
    assign('resred', 1, env = .GlobalEnv)
    setwd(tclvalue(path))
    dat = readBin(paste(namebase,
tomaxdigs(as.numeric(tclvalue(firstnum))), nameext, sep
= ''),what='raw',n=bytes,size=1)
    lenraw = length(dat)
    totpixels=imxdim*imydim
```

```
    headerplus1=lenraw-totpixels+1
    ymat=matrix(as.double(dat[headerplus1:
lenraw]),nrow=imxdim)
    xmat=ymat[,imydim:1]
    assign('xmat', xmat, env = .GlobalEnv)
    tkdestroy(tt)
    crop()}

  OK.but <-tkbutton(tt,text='   OK   ',command=OnOK)
  tkbind(entryname, '<Return>',OnOK)#???
  tkgrid(OK.but)
  tkfocus(tt)}
```

Crop Image
The user crops the first image by first clicking the point that
marks the bottom/left boundary of the image and then the
top/right. The same cropping is applied to all subsequent
images. Cropping can dramatically reduce computation time.

```
crop = function() {

  customimage(xmat, 'Original Image', F, 1)
  tkmessageBox(message = 'Please crop the image')
  croplocs = myloc(2, xmat, 1)
  xmat = xmat[loc[1,1]:loc[2,1], loc[1,2]:loc[2,2]]
  assign('croplocs1', c(loc[1,1], loc[2,1]), env
= .GlobalEnv)
  assign('croplocs2', c(loc[1,2], loc[2,2]), env
= .GlobalEnv)
  assign('xmat', xmat, env = .GlobalEnv)
  customimage(xmat, 'Cropped Image', T, 1)
  choosetip()}
```

Choose tip
The user chooses (by clicking on the image) an arbitrary point
which will defines the base of the tip (a reference points from
which the growth measurements will be made), and then
chooses the direction in which growth will be measured.

```
choosetip = function() {
```

```
  tkmessageBox(message = 'Please choose a point that
marks the beginning of the tip')

  base = myloc(1, xmat, 1)

  tkmessageBox(message = 'Please choose a point to
indicate the direction of growth')

  direct = myloc(1, xmat, 1)

  lines(x = c(base[1], direct[1])*mpp*resred, y =
c(base[2], direct[2])*mpp*resred, col = 'green')

  slope = (direct[2]-base[2])/(direct[1]-base[1])

  abline((base[2] + (base[1]-1)/slope)*mpp*resred, -1/
slope, col = 'green')

  assign('base', base, env = .GlobalEnv)
  assign('direct', direct, env = .GlobalEnv)

  prenewthresh()}
```

```
# The following functions help the user to convert an (often messy)
# grayscale image of a stolon into a binary one: areas deemed part
# of the stolon are assigned the value 1, and other areas are
# assigned the value 0.  This is done in the following manner:

# First we apply a grayscale threshold to the image.  All
# brightness values less than the threshold value are
# displayed in white in the first image displayed.

# The user applies a threshold to the gradient of the brightness
# values.  If the maximum brightness difference from one pixel
# to one of its neighbors is greater than the threshold, then point
# is displayed in white in the second image.

# A new (third) image is displayed where a pixel is given the
# value 1 if it has the value 1 in either of the first two images.
```

This resulting (still messy) image/matrix is further ‘cleaned’
using the tools `regrow` and `erode`.

`Erode(x)` turns foreground points with X or more background
neighbors to background points.

`Regrow(x)` turns background points with X or more foreground
neighbors to foreground points.

Next, we consider only the largest contiguous white (1-valued)
area in the image. All other 1-valued points are set to 0. The
image is almost ready for analysis. The last step (circle
algorithm) is described below.

Brightness/gradient threshold

```
prenewthresh = function() {

  maxchange = matrix(nrow = nrow(xmat)-2, ncol =
ncol(xmat)-2)

  for(i in 1:nrow(maxchange)) {

    for(j in 1:ncol(maxchange)) {

      maxchange[i,j] = max(xmat[i:(i+2), j:(j+2)] -
xmat[i+1, j+1])}}

  newmaxchange = xmat
  newmaxchange[,] = 0
  newmaxchange[2:(nrow(xmat)-1), 2:(ncol(xmat)-1)] =
maxchange
  maxchange = newmaxchange

  assign('maxchange', maxchange, env = .GlobalEnv)

  quartz(width = 3, height = 9)
  assign('bt', 80, env = .GlobalEnv)
  assign('gt', 10, env = .GlobalEnv)
```

```
  split.screen( figs = c( 3, 1 ), erase = F)
  screen(1, new = F)
  customimage(xmat < 80, 'Brightness', T, 1)
  screen(2)
  customimage(maxchange > 10, 'Gradient', T, 1)
  screen(3)
  customimage(((xmat < 80) + (maxchange > 10)) > 0,
'Combo', T, 1)
  newthresh()}

newthresh = function() {

  tv = tclVar(bt)
  gv = tclVar(gt)

  plustenB = function() {assign('bt', bt + 10, envir
= .GlobalEnv)
    tclvalue(tv) = bt
    refresh(1)}
  minustenB = function() {assign('bt', bt - 10, envir
= .GlobalEnv)
    refresh(1)
    tclvalue(tv) = bt}
  plusoneB = function() {assign('bt', bt + 1, envir
= .GlobalEnv)
    refresh(1)
    tclvalue(tv) = bt}
  minusoneB = function() {assign('bt', bt - 1, envir
= .GlobalEnv)
    refresh(1)
    tclvalue(tv) = bt}

  plusoneG = function() {assign('gt', gt + 1, envir
= .GlobalEnv)
    refresh(2)
    tclvalue(gv) = gt}
  minusoneG = function() {assign('gt', gt - 1, envir
= .GlobalEnv)
    refresh(2)
    tclvalue(gv) = gt}
```

```
  refresh = function(x) {
    screen(x)
    if(x==1) customimage(xmat < bt, 'Brightness', T, 1)
    if(x==2) customimage(maxchange > gt, 'Gradient', T,
1)
    screen(3)
    customimage(((xmat < bt) + (maxchange > gt)) > 0,
'Combo', T, 1) }

  enterB = function() {
    assign('bt', as.numeric(tclvalue(tv)), env
= .GlobalEnv)
    screen(1)
    customimage(xmat < bt, 'Brightness', T, 1)
    screen(3)
    customimage(((xmat < bt) + (maxchange > gt)) > 0,
'Combo', T, 1)}

  enterG = function() {
    assign('gt', as.numeric(tclvalue(gv)), env
= .GlobalEnv)
    screen(2)
    customimage(maxchange > gt, 'Gradient', T, 1)
    screen(3)
    customimage(((xmat < bt) + (maxchange > gt)) > 0,
'Combo', T, 1)}

  OnOK <- function() {
    tkdestroy(tt)
    assign('bwmat', ((xmat < bt) + (maxchange > gt)) >
0, env = .GlobalEnv)
    assign('bwmatorig', bwmat, env = .GlobalEnv)
    tkmessageBox(message = 'Please clean the image')
    dev.off()
    customimage(bwmat, '', F, 1)
    assign('brightnessthresh', bt, env = .GlobalEnv)
    assign('gradientthresh', gt, env = .GlobalEnv)
    assign('cleaningsteps', array(dim = c(0, 2)), env
= .GlobalEnv)
    cleaning()}
```

```
  tt <- tktoplevel()

  entryt = tkentry(tt, width = '5', textvariable = tv)
  entryg = tkentry(tt, width = '5', textvariable = gv)

  ptb = tkbutton(tt, text = 'plusten brightness',
command = plustenB)
  mtb = tkbutton(tt, text = 'minusten brightness',
command = minustenB)
  pob = tkbutton(tt, text = 'plusone brightness',
command = plusoneB)
  mob = tkbutton(tt, text = 'minusone brightness',
command = minusoneB)
  enterb.but = tkbutton(tt,text='  Enter
',command=enterB)
  pog = tkbutton(tt, text = 'plusone gradient', command
= plusoneG)
  mog = tkbutton(tt, text = 'minusone gradient',
command = minusoneG)
  enterg.but = tkbutton(tt,text='  Enter
',command=enterG)
  OK.but <-tkbutton(tt,text='   OK   ',command=OnOK)

  tkgrid(ptb)
  tkgrid(mtb)
  tkgrid(pob)
  tkgrid(mob)
  tkgrid(entryt)
  tkgrid(enterb.but)
  tkgrid(pog)
  tkgrid(mog)
  tkgrid(entryg)
  tkgrid(enterg.but)
  tkgrid(OK.but)}
```

Image Cleaning

```
cleaning = function() {

  tt <- tktoplevel()
```

Define erode()

```
  preerode = function() {

    tkmessageBox(message='Enter X')

    tt2 = tktoplevel()
    tv = tclVar()
    nn = tkentry(tt2, width = 5, textvariable = tv)

    OnOK = function() {

      if(!tclvalue(tv) %in% 2:8) {print('Try harder,
Noah'); tkdestroy(tt2)} else {
        erode(bwmat, tclvalue(tv))
        tkdestroy(tt2)}}

  tkgrid(nn)
  Ok.but = tkbutton(tt2, text = 'OK', command = OnOK)
  tkgrid(Ok.but)}

  erode = function(mat, n) {
```

Do corners and border points first

```
    if(!prod(dim(mat)==dim(bwmat)) || prod(mat!=bwmat))
R = T else R = F

    tempmat = mat; tempmat[,] = 0

    tempmat[(1+1):(nrow(mat)-1), (1+1):(ncol(mat)-1)] =
mat[1:(nrow(mat)-2), 1:(ncol(mat)-2)]+
                                    mat[1:
(nrow(mat)-2), (1+1):(ncol(mat)-1)]+
                                    mat[1:
(nrow(mat)-2), (1+2):ncol(mat)]+
                                    mat[(1+1):
(nrow(mat)-1), 1:(ncol(mat)-2)]+
                                    mat[(1+1):
(nrow(mat)-1), (1+2):ncol(mat)]+
```

```
mat[(1+2):nrow(mat), 1:(ncol(mat)-2)]+

mat[(1+2):nrow(mat), (1+1):(ncol(mat)-1)]+

mat[(1+2):nrow(mat), (1+2):ncol(mat)]

    if(R) return((mat + (tempmat >= n)) == 2) else {

      assign('save', mat, env = .GlobalEnv)
      assign('bwmat', (mat + (tempmat >= n)) == 2, env
= .GlobalEnv)
      customimage(bwmat, main = 'Image Cleaning', T, 1)
      if(!exists('firstrun')) assign('cleaningsteps',
rbind(cleaningsteps, as.numeric(c(1, n))), env
= .GlobalEnv)}}
```

Define regrow()

```
  preregrow = function() {

    tkmessageBox(message='Enter X')

    tt2 = tktoplevel()
    tv = tclVar()
    nn = tkentry(tt2, width = 5, textvariable = tv)

    OnOK = function() {

      if(!tclvalue(tv) %in% 2:8) {print('Try harder,
Noah'); tkdestroy(tt2)} else {
        regrow(bwmat, tclvalue(tv))
        tkdestroy(tt2)}}

  tkgrid(nn)
  Ok.but = tkbutton(tt2, text = 'OK', command = OnOK)
  tkgrid(Ok.but)}

  regrow = function(mat, n) {
```

Do corners and border points first

```
    if(!prod(dim(mat)==dim(bwmat)) || prod(mat!=bwmat))
R = T else R = F

    tempmat = mat; tempmat[,] = 0

    tempmat[(1+1):(nrow(mat)-1), (1+1):(ncol(mat)-1)] =
mat[1:(nrow(mat)-2), 1:(ncol(mat)-2)]+
                                    mat[1:
(nrow(mat)-2), (1+1):(ncol(mat)-1)]+
                                    mat[1:
(nrow(mat)-2), (1+2):ncol(mat)]+
                                    mat[(1+1):
(nrow(mat)-1), 1:(ncol(mat)-2)]+
                                    mat[(1+1):
(nrow(mat)-1), (1+2):ncol(mat)]+

mat[(1+2):nrow(mat), 1:(ncol(mat)-2)]+

mat[(1+2):nrow(mat), (1+1):(ncol(mat)-1)]+

mat[(1+2):nrow(mat), (1+2):ncol(mat)]

    if(R) return((mat + (tempmat >= n)) > 0) else {

      assign('save', mat, env = .GlobalEnv)
      assign('bwmat', (mat + (tempmat >= n)) > 0, env
= .GlobalEnv)
      customimage(bwmat, main = 'Image Cleaning', T, 1)

      if(!exists('firstrun')) assign('cleaningsteps',
rbind(cleaningsteps, as.numeric(c(2, n))), env
= .GlobalEnv)}}
```

`doubledisplay` shows the original and processed images simultaneously
in a chessboard type pattern, allowing the user to check his results.

```
doubledisplay = function() {

  mat1 = xmat
  mat2 = bwmat
```

```
  n = 10

  mat1 = mat1*255/max(mat1)
  mat2 = mat2*255/max(mat2)

  display = array(0, dim = dim(mat1))

  for(i in 1:n) {

    for(j in 1:n) {

      if((i-j) %% 2 == 1) display[((i-1)/
n*nrow(mat1)+1): (i/n*nrow(mat1)), ((j-1)/
n*ncol(mat1)+1): (j/n*ncol(mat1))] =
      mat1[((i-1)/n*nrow(mat1)+1): (i/n*nrow(mat1)),
((j-1)/n*ncol(mat1)+1): (j/n*ncol(mat1))]

      if((i-j) %% 2 == 0) display[((i-1)/
n*nrow(mat1)+1): (i/n*nrow(mat1)), ((j-1)/
n*ncol(mat1)+1): (j/n*ncol(mat1))] =
      mat2[((i-1)/n*nrow(mat1)+1): (i/n*nrow(mat1)),
((j-1)/n*ncol(mat1)+1): (j/n*ncol(mat1))]}}

  customimage(display, "", T, 1)}

  goback = function() {

    assign('bwmat', save, env = .GlobalEnv)
    customimage(bwmat, main = 'Thresholded Image', T,
1)
    if(nrow(cleaningsteps)==1) assign('cleaningsteps',
array(dim = c(0, 2)), env = .GlobalEnv) else
assign('cleaningsteps', cleaningsteps[1:
(nrow(cleaningsteps)-1),], env = .GlobalEnv)}

  loadorig = function() {

    assign('bwmat', bwmatorig, env = .GlobalEnv)
    customimage(bwmat, main = 'Thresholded Image', T,
1)
    assign('cleaningsteps', array(dim = c(0, 2)), env
```

```
= .GlobalEnv)}

  OnOK = function() {

    tkdestroy(tt)

    dev.off()
    customimage(xmat, main = '', T, 1)
    assign('erode', erode, env = .GlobalEnv)
    assign('regrow', regrow, env = .GlobalEnv)
    extractshape()}

  er = tkbutton(tt, text = 'Erode: Set foreground
points with X or more background neighbors to
background points',
    command = preerode)
  rg = tkbutton(tt, text = 'Regrow: Set background
points with X or more foreground neighbors to
foreground points',
    command = preregrow)
  gb = tkbutton(tt, text = 'Undo previous step',
command = goback)
  dd = tkbutton(tt, text = 'Display both images',
command = doubledisplay)
  or = tkbutton(tt, text = 'Restart from original
thresholded image', command = loadorig)
  ok = tkbutton(tt, text = 'Done',  command = OnOK)

  tkgrid(er)
  tkgrid(rg)
  tkgrid(gb)
  tkgrid(dd)
  tkgrid(or)
  tkgrid(ok)}
```

Extract shape

```
extractshape = function() {

  assign('resred2', 3, env = .GlobalEnv)
```

```
  newx = floor(nrow(bwmat)/resred2)
  newy = floor(ncol(bwmat)/resred2)

  cut = bwmat[1:(resred2*newx),1:(resred2*newy)]
  avs = array(dim = c(newx, newy))

  for(i in 1:newx) {
    for(j in 1:newy) {
      avs[i,j] = mean(cut[(resred2*i-(resred2-1)):
(resred2*i),((resred2*j-(resred2-1)):(resred2*j))]) }}

  smallbwmat = avs > .5

  newbwmat = array(0, dim = dim(smallbwmat)+c(2,2))
  newbwmat[2:(nrow(newbwmat)-1),2:(ncol(newbwmat)-1)] =
smallbwmat
  singlepiece = newbwmat
  singlepiece[,] = 0
  maxsum = 0

  for(i in 1:nrow(newbwmat)) {
    for(j in 1:ncol(newbwmat)) {
      if(newbwmat[i,j] == 1) {

      singlepiece = newbwmat; singlepiece[] = 0
      singlepiece[i,j] = 1
      poschanges = singlepiece
      lastsum = 0

      while(1) {

        poschanges[(1+1):(nrow(singlepiece)-1), (1+1):
(ncol(singlepiece)-1)] = singlepiece[1:
(nrow(singlepiece)-2),
         1:(ncol(singlepiece)-2)]+
               singlepiece[1:(nrow(singlepiece)-2),
(1+1):(ncol(singlepiece)-1)]+
               singlepiece[1:(nrow(singlepiece)-2),
(1+2):ncol(singlepiece)]+
               singlepiece[(1+1):(nrow(singlepiece)-1),
1:(ncol(singlepiece)-2)]+
```

```
              singlepiece[(1+1):(nrow(singlepiece)-1),
(1+1):(ncol(singlepiece)-1)]+
              singlepiece[(1+1):(nrow(singlepiece)-1),
(1+2):ncol(singlepiece)]+
              singlepiece[(1+2):nrow(singlepiece), 1:
(ncol(singlepiece)-2)]+
              singlepiece[(1+2):nrow(singlepiece),
(1+1):(ncol(singlepiece)-1 )]+
              singlepiece[(1+2):nrow(singlepiece),
(1+2):ncol(singlepiece)]

        poschanges = poschanges > 0
        singlepiece = (poschanges + newbwmat) == 2

        if(lastsum == sum(singlepiece)) {
          newbwmat = newbwmat - singlepiece
          if(lastsum > maxsum) {biggestpiece =
singlepiece; maxsum = lastsum}
          break()}

        lastsum = sum(singlepiece) }}}}

  biggestpiece = biggestpiece[2:(nrow(biggestpiece)-1),
2:(ncol(biggestpiece)-1)]

  newbwmat = -biggestpiece + 1

  newbiggestpiece = array(0, dim = dim(newbwmat))
  if(!biggestpiece[2, 2]) newbiggestpiece[2, 2] = 1
  if(!biggestpiece[2, ncol(newbiggestpiece)-1])
newbiggestpiece[2, ncol(newbiggestpiece)-1] = 1
  if(!biggestpiece[nrow(biggestpiece)-1, 2])
newbiggestpiece[nrow(biggestpiece)-1, 2] = 1
  if(!biggestpiece[nrow(biggestpiece)-1,
ncol(newbiggestpiece)-1])
newbiggestpiece[nrow(biggestpiece)-1,
ncol(newbiggestpiece)-1] = 1
  sum = 1

  while(1) {
```

```
    lastsum = sum

    poschanges = array(0, dim = dim(newbiggestpiece))

    poschanges[(1+1):(nrow(newbiggestpiece)-1), (1+1):
(ncol(newbiggestpiece)-1)] = newbiggestpiece[1:
(nrow(biggestpiece)-2),
      1:(ncol(biggestpiece)-2)]+
                                              newbiggestpiece[1:
(nrow(biggestpiece)-2), (1+1):(ncol(biggestpiece)-1)]+
                                              newbiggestpiece[1:
(nrow(biggestpiece)-2), (1+2):ncol(biggestpiece)]+

newbiggestpiece[(1+1):(nrow(biggestpiece)-1), 1:
(ncol(biggestpiece)-2)]+

newbiggestpiece[(1+1):(nrow(biggestpiece)-1), (1+1):
(ncol(biggestpiece)-1)]+

newbiggestpiece[(1+1):(nrow(biggestpiece)-1),
(1+2):ncol(biggestpiece)]+

newbiggestpiece[(1+2):nrow(biggestpiece), 1:
(ncol(biggestpiece)-2)]+

newbiggestpiece[(1+2):nrow(biggestpiece), (1+1):
(ncol(biggestpiece)-1 )]+

newbiggestpiece[(1+2):nrow(biggestpiece),
(1+2):ncol(biggestpiece)]

    poschanges = poschanges > 0

    newbiggestpiece = (poschanges + newbwmat) == 2
    sum = sum(newbiggestpiece, na.rm = T)

    if(sum == lastsum) break()}

  newbiggestpiece[1,] = 1
  newbiggestpiece[,1] = 1
  newbiggestpiece[nrow(newbiggestpiece),] = 1
```

```
  newbiggestpiece[,ncol(newbiggestpiece)] = 1

  biggestpiece = - newbiggestpiece + 1

#Some extra smoothing is applied.

  biggestpiece = erode(biggestpiece, 5)
  biggestpiece = regrow(biggestpiece, 4)

  assign('biggestpiece', biggestpiece, env
 = .GlobalEnv)

  smooth()}
```

Circle algorithm:

One final method for smoothing is applied before we analyze the image.
It works primarily on the edges.

The ‘circle algorithm’ starts at the point selected as the base of the tip
and moves toward the growing end in a straight line 5 pixels at a time.
Call this line X.

At every 5 pixel stop along X, we examine circles that are centered
on the line perpendicular to X, up to 5 pixels away from X on either side.

At each point, we find the largest circle containing at least the
proportion p (.9985) foreground points. All values of the largest circle
for each stop on X are be deemed foreground points and other points
# of the tip not enclosed by any circles now become background points.

```
biggestcircle = function(mat, x, y, p) {

  r = 5

  while(1) {

    circle = array(0, dim = dim(biggestpiece))

    for(i in max(0, (x-r)):min(nrow(circle), (x+r)))
 for(j in max(0, (y-r)):min(ncol(circle),
```

```
      (y+r))) if(((i-x)^2+(j-y)^2)<r^2) circle[i,j] = 1

    if(sum((circle+biggestpiece)==2) < (p *
 sum(circle))) return(circle)

    r = r + 2 }}

smooth = function() {

  p = 0.9985
  dirx = (direct-base)/sqrt(sum((direct-base)^2))
  diry = c(dirx[2], -dirx[1])
  circles = array(0, dim = dim(biggestpiece))

  x0 = round(base[1]/3)
  y0 = round(base[2]/3)

  while(1) {

    sum = 0
    x = x0
    y = y0

    while(1) {

      newbiggest = biggestcircle(biggestpiece,
 round(x), round(y), p)

      if(sum(newbiggest) < sum) break()

      sum = sum(newbiggest)

      x = x + diry[1]; y = y + diry[2]

      biggest1 = newbiggest }

    sum = 0
    x = x0
    y = y0

    while(1) {
```

```
      newbiggest = biggestcircle(biggestpiece,
round(x), round(y), p)

      if(sum(newbiggest) < sum) break()

      sum = sum(newbiggest)

      x = x - diry[1]; y = y - diry[2]

      biggest2 = newbiggest }

    if(sum(biggestpiece+biggest1==2) > sum(biggestpiece
+biggest2==2)) circles = circles +
      biggest1 else circles = circles + biggest2

    x0 = x0 + 5*dirx[1]
    y0 = y0 + 5*diry[1]

    if(max(sum(biggest1), sum(biggest2)) < 800)
break()}

  assign('biggestpiece', circles>0, env = .GlobalEnv)
  findstats()}
```

\# Calculate stats
\# Now that the process of converting the image to a binary one is
\# complete, we compute the stolon length at angles (-pi/4, -pi/6,
\# -pi/12, 0, pi/12, pi/6, pi/4) from the line X. These distances and
\# the area of the stolon tip are saved to the matrix `stats`.

```
findstats = function() {

  angles = c(-pi/4, -pi/6, -pi/12, 0, pi/12, pi/6, pi/
4)

  if(!exists('stats')) stats = array(dim = c(0,
length(angles)+1))

  testarea = biggestpiece
  testarea[,] = 0
```

```
  slope = -1/((direct[2]-base[2])/(direct[1]-base[1]))
  origangle = findangle(base, direct)
  yint = ((base[2] + (base[1]-1)/(-1/slope)))/resred/
resred2

  if((sin(origangle)-base[2])>0) {

    for(i in 1:nrow(biggestpiece)) {

      if((yint + slope*i) > .5 && (yint + slope*i) <
(ncol(biggestpiece)+.5)) {

        testarea[i, round(yint +
slope*i):ncol(biggestpiece)] = 1}

      if((yint + slope*i) < .5) testarea[i,
1:ncol(biggestpiece)] = 1}}

  if((sin(origangle)-base[2])<0) {

    for(i in 1:nrow(biggestpiece)) {

      if((yint + slope*i) > .5 && (yint + slope*i) <
(ncol(biggestpiece)+.5)) {

        testarea[i, 1:round(yint + slope*i)] = 1}

      if((yint + slope*i) > (ncol(biggestpiece)+ .5))
testarea[i, 1:ncol(biggestpiece)] = 1}}

  area = sum(testarea
+biggestpiece==2)*resred^2*mpp^2*resred2^2

  curpoint = round(base/resred2)

  lengths = numeric(length(angles))

  for(i in 1:length(angles)) {

    curpoint = round(base/resred2)
```

```
    ang = origangle + angles[i]

    len = 0

    while(1) {

      curpoint = curpoint+c(cos(ang), sin(ang))

      len = len + 1

      if(!biggestpiece[round(curpoint[1]),
 round(curpoint[2])]) break()}

    lengths[i] = len*resred*mpp*resred2}

  assign('stats', rbind(stats, c(area, lengths)), env
 = .GlobalEnv)
  saveimage()
  if(!exists('firstrun')) looprest()}
```

`saveimage` creates a jpeg of the original stolon tip image with
boundary bounds displayed in green and growth direction
displayed it blue.

```
saveimage = function() {

  sums = biggestpiece; sums[]=0

  sums[2:(nrow(sums)-1), 2:(ncol(sums)-1)] =
  biggestpiece[1:(nrow(biggestpiece)-2), 1:
 (ncol(biggestpiece)-2)] +
  biggestpiece[1:(nrow(biggestpiece)-2), 2:
 (ncol(biggestpiece)-1)] +
  biggestpiece[1:(nrow(biggestpiece)-2), 3:
 ncol(biggestpiece)] +
  biggestpiece[2:(nrow(biggestpiece)-1), 1:
 (ncol(biggestpiece)-2)] +
  biggestpiece[2:(nrow(biggestpiece)-1), 2:
 (ncol(biggestpiece)-1)] +
  biggestpiece[2:(nrow(biggestpiece)-1), 3:
 ncol(biggestpiece)] +
```

```
  biggestpiece[3:nrow(biggestpiece), 1:
(ncol(biggestpiece)-2)] +
  biggestpiece[3:nrow(biggestpiece), 2:
(ncol(biggestpiece)-1)] +
  biggestpiece[3:nrow(biggestpiece), 3:
ncol(biggestpiece)]

  perim = sums<9 & biggestpiece == 1

  mkrowcol(perim)
  rows = rows*resred2
  cols = cols*resred2

  if(!exists('picnum')) picnum = num1
  jpeg(tomaxdigs(picnum), width = 2000, height = 2000,
type = "cairo")

  image(xmat, main = picnum)

  for(i in 1:3) points(rows/nrow(xmat), cols/
ncol(xmat), col = 'green', cex = 3)

  lines(x = c(base[1], direct[1])/nrow(xmat), y =
c(base[2], direct[2])/ncol(xmat), col = 'blue', lwd =
3)

  slope = (direct[2]-base[2])/(direct[1]-
base[1])*(nrow(xmat)/ncol(xmat))

  abline((base[2]/ncol(xmat) + (base[1]-1)/nrow(xmat)/
slope), -1/slope, col = 'blue', lwd = 3)

  dev.off()}
```

```
# The same cropping, cleaning, and analysis steps are now
# applied to every other image in the time series.  This could
# take some time!

looprest = function() {

  assign('firstrun', F, env = .GlobalEnv)
```

loop through all images

```
  for(i in (num1+1):num2) {

    print(i/num2)
    assign('picnum', i, env = .GlobalEnv)
```

read file

```
    filename = paste(namebase, tomaxdigs(i), nameext,
sep = '')

    dat = readBin(filename,what='raw',n=bytes,size=1)
    lenraw = length(dat)
    totpixels=imxdim*imydim
    headerplus1=lenraw-totpixels+1
    ymat=matrix(as.double(dat[headerplus1:
lenraw]),nrow=imxdim)
    xmat=ymat[,imydim:1]
```

crop file

```
    xmat = xmat[croplocs1[1]:croplocs1[2],
croplocs2[1]:croplocs2[2]]
```

threshold image

```
    maxchange = matrix(nrow = nrow(xmat)-2, ncol =
ncol(xmat)-2)

    for(k in 1:nrow(maxchange)) {

      for(j in 1:ncol(maxchange)) {

        maxchange[k,j] = max(xmat[k:(k+2), j:(j+2)] -
xmat[k+1, j+1])}}

    newmaxchange = xmat
    newmaxchange[,] = 0
    newmaxchange[2:(nrow(xmat)-1), 2:(ncol(xmat)-1)] =
```

```
maxchange
    maxchange = newmaxchange

    bwmat = ((xmat < bt) + (maxchange > gt)) > 0

    assign('bwmat', bwmat, env = .GlobalEnv)
    assign('xmat', xmat, env = .GlobalEnv)
```

clean image

```
    for(j in 1:nrow(cleaningsteps)) {

      if (cleaningsteps[j, 1] == 1) erode(bwmat,
cleaningsteps[j,2]) else regrow(bwmat, cleaningsteps[j,
2]) }

    extractshape()
    saveimage()}}

firstplot = T
assign('oldpoint', 1, env = .GlobalEnv)

#begin()
```
