# Supplementary material for: The Selective Myosin II Inhibitor Blebbistatin Reversibly Eliminates Gastrovascular Flow and Stolon Tip Pulsations in the Colonial Hydroid Podocoryna carnea
Source: PLoS One. 2015 Nov 25;10(11):e0143564. doi: 10.1371/journal.pone.0143564 (PMC4659590; doi:10.1371/journal.pone.0143564)
Supplement: S2 Software — (DOCX) [file pone.0143564.s006.docx]

# R code to read Axiovision csv file output of profiles and convert

# to time dependent images

secperframe=8 # user specifies the frame rate

mpp=0.1573 # user specifies the number of microns per pixel

# specify the frames of the csv file to plot

framechoice=c(200:800)

# choose and output file name

pdfout="Polyp-bud-bleb-test3.pdf"

# specify the path to the folder where the csv file resides

setwd("/Users/...")

# specify the file name to read

filename='29 sept isolated tip part 1.csv'

# read the file into dat, and extract the position number, x and y locations

# from columns 1, 2, and 3

dat = read.csv(filename, skip = 1)

loc <- dat[,1]

xloc <- dat[,2]

yloc <- dat[,3]

# convert these into distances along the prifile

xlocref<-xloc[1]

ylocref<-yloc[1]

distpix<-xloc # to dimension

distpix<-sqrt((xloc-xlocref)^2 + (yloc-ylocref)^2)

xreldist=(xloc-xloc[1])*mpp # convert pixel rel. dist to microns

yreldist=(yloc-yloc[1])*mpp # convert pixel rel. dist to microns

distmicron<-xloc # to dimension only

distmicron<-sqrt((xreldist)^2 + (yreldist)^2)

# extract the time dependent gray values from the remaining columns

dattemp = as.matrix(dat[,-c(1, 2, 3)])

# take the transpose of the dattemp to make time in rows, distance in columns

tdat=t(dattemp)

#BLEB TEST

image(framechoice*secperframe,distmicron,tdat[framechoice,], col = gray((1:320)/320),

xlab="Time (sec)",ylab="Distance (microns)")

# the start time can be modified if necessary, or divided by 60 to show time in minutes

# write a pdf file to file name pdfout

dev.copy2pdf(file=pdfout)
